# Regulation of forest defoliating insects through small mammal predation: reconsidering the mechanisms

**DOI:** 10.1007/s00442-014-3080-x

**Published:** 2014-09-19

**Authors:** Ida Kollberg, Helena Bylund, Otso Huitu, Christer Björkman

**Affiliations:** 1 Department of Ecology, Swedish University of Agricultural Sciences, Box 7044, 750 07 Uppsala, Sweden; 2 Finnish Forest Research Institute (Metla), Suonenjoki, Finland

**Keywords:** Herbivore, Functional response, Natural enemies, Outbreaks, Population dynamics

## Abstract

Population densities of forest defoliating insects may be regulated by small mammal predation on the pupae. When outbreaks do occur, they often coincide with warm, dry weather and at barren forest sites. A proposed reason for this is that weather and habitat affect small mammal population density (numerical response) and hence pupal predation. We propose an alternative explanation: weather and habitat affect small mammal feeding behaviour (functional response) and hence the outbreak risks of forest pest insects. We report results from laboratory and field-enclosure experiments estimating rates of pupal predation by bank voles (*Myodes glareolus*) on an outbreak insect, the European pine sawfly (*Neodiprion sertifer*), at different temperatures (15 and 20 °C), in different microhabitats (sheltered and non-sheltered), and with or without access to alternative food (sunflower seeds). We found that the probability of a single pupa being eaten at 20 °C was lower than at 15 °C (0.49 and 0.72, respectively). Pupal predation was higher in the sheltered microhabitat than in the open one, and the behaviour of the voles differed between microhabitats. More pupae were eaten in situ in the sheltered microhabitat whereas in the open area more pupae were removed and eaten elsewhere. Access to alternative food did not affect pupal predation. The results suggest that predation rates on pine sawfly pupae by voles are influenced by temperature- and habitat-induced variation in the physiology and behaviour of the predator, and not necessarily solely through effects on predator densities as previously proposed.

## Introduction

The density of herbivorous insect populations is affected by mortality caused by different kinds of natural enemies. Mortality rates in predator–prey interactions are primarily determined by the density of the prey and the density of the predator (Holling 1961). The regulation of prey populations often requires predators to exhibit a numerical response as typically seen in specialist predators and parasitoids. Generalist predators normally do not show as strong a numerical response to a specific prey as specialist predators but their densities are rather influenced by the abundance of all prey in the habitat (Murdoch et al. 1985). Nonetheless, generalist predators have the capacity to regulate low-density insect populations through strong functional responses (Elkinton et al. 1996; Parry et al. 1997; Tanhuanpää et al. 1999). Thus, to understand how predation affects prey populations it is important to determine the type of predator involved and which mechanisms affect their numerical and functional responses.

Generalist small mammals have the potential to be involved in the regulatory process of forest defoliating insect populations because, among other food items, they feed on pupae buried in the ground (East 1974; Walsh 1990; Cook et al. 1994). For example, it has been suggested that small mammals—shrews, mice and voles—control populations of the forest pests the European pine sawfly (*Neodiprion sertifer*) and the gypsy moth (*Lymantria* *dispar*) during their endemic phases (Holling 1959; Hanski and Parviainen 1985; Olofsson 1987; Elkinton et al. 1996, but see Liebhold et al. 2000). But occasionally insect populations escape regulation and rapidly increase in density, resulting in what is commonly referred to as an ‘outbreak’. One suggested explanation for forest defoliating insect outbreaks is a relaxation of the small mammal predation pressure on the pupae (Holling 1959; Hanski and Parviainen 1985).

It is widely acknowledged that forest insect densities are affected by weather, either due to direct effects on the herbivore (Neuvonen et al. 1999; Bale et al. 2002; Soubeyrand et al. 2008) or indirect effects through the host plant or natural enemies (Virtanen and Neuvonen 1999; Joern et al. 2006). However, the mechanisms behind how weather influences outbreak dynamics are not well understood. The anticipated role of climate in periodic insect outbreak species has been rejected since climatic variation is assumed to be of a more chaotic nature (Martinat 1987). Nevertheless, climate warming has led to the disruption of periodicity in some species, caused by not easily foreseen effects of biotic and abiotic interactions (Johnson et al. 2010). In species showing more irregular eruptive outbreak patterns, however, weather may play an important role for initiating outbreaks. Outbreaks of the European pine sawfly, for example, occur highly irregularly (Kolomiets et al. 1979) and often start after a series of warm and dry summers (McLeod 1970; Kolomiets et al. 1979). Although the association between weather and sawfly outbreaks has not been tested statistically due to lack of long-term data, it is generally believed that anomalies in weather are responsible for changes in insect abundances. A reason suggested for sawfly outbreaks is that warm and dry conditions lead to reduced reproduction in small mammals due to decreased access to food, which leads to lower densities and lower predation pressure on insect pupae (Pankakoski 1985; Hanski and Parviainen 1985). An alternative explanation is that temperature affects the metabolism of the small mammals and hence their appetite and functional response. Mammalian predators are likely to decrease their food intake when temperatures increase because they need less energy to maintain their body temperature (Sibly 1981). As an explanation for outbreaks, this modified small mammal predation hypothesis, suggesting that the functional responses are affected by changed environmental conditions, has not been tested.

Apart from the connection to weather conditions, it is also often observed that sawfly outbreaks occur in forests growing on nutrient-poor soils (McLeod 1970; Kolomiets et al. 1979). Again, the reason may be that the abundance of small mammals and hence the pupal predation pressure is influenced by forest type (Hanski and Parviainen 1985; Herz and Heitland 2003). Forests on nutrient-rich sites are normally inhabited by larger populations of small mammals than forests on more impoverished sites (Hanski and Parviainen 1985), particularly during the critical low-density phase of the small mammal population cycle (Hansson 1969). Food resources are generally more abundant in rich forest habitats, and this has a direct positive influence on small mammal populations. A rich forest also usually contains more sheltering structures (Ecke et al. 2002), which small mammals use to escape predators (Kotler et al. 1991; Korpimäki et al. 1996).

It is also reasonable to assume that forest type affects the foraging activity of each predator individual. It is, for example, well known that microhabitat influences the foraging behaviour of small mammals (Kotler et al. 1991; Korpimäki et al. 1996) and that alternative food resources affect their foraging decisions (Elkinton et al. 2004). Two specific predictions can be made concerning patch quality and availability of food resources: individuals should stop foraging in a patch when the harvesting benefits fall below the foraging costs (Charnov 1976; Brown 1988); and predation on pupae should decrease when an alternative food source of higher quality is available (Holling 1965; Murdoch 1969). Hence, the structure of the forest floor in combination with availability of food resources may influence the foraging behaviour of small mammals significantly and, consequently, affect the predation pressure and influence the risk of outbreaks in certain insect species.

To summarise, we have identified three factors—temperature, microhabitat and alternative food sources—with the potential to affect the functional response of small mammals through changes in their metabolism and feeding behaviour. To quantify how these factors affect the predation rates of small mammals, we conducted laboratory experiments at two temperatures to study the metabolic effect, and experiments in field enclosures to evaluate the role of microhabitat and alternative food on vole behaviour. As model species we used the European pine sawfly (*Neodiprion sertifer*) which is one of the most well-studied outbreak insect species and on which many of the original empirical studies and theoretical papers about outbreaks are based, and the bank vole (*Myodes glareolus*) a known predator of *N. sertifer*. The hypotheses tested were:

1. Predation by voles on sawfly pupae is inversely related to temperature.
2. Voles prefer to occupy and consume prey in sheltered, ‘non-risky’, microhabitats as compared to open, ‘risky’, habitats.
3. When voles have access to alternative food, pupal predation is more reduced in open habitats than in sheltered habitats.

The aim of the study was to increase our knowledge of how temperature and habitat conditions affect the rate of pupal predation by small mammals and hence the likelihood of pine sawfly outbreaks.

## Materials and methods

### Study species

The European pine sawfly, *Neodiprion sertifer* Geoffr. (Hymenoptera: Diprionidae), is a univoltine outbreak species that feeds on pine (*Pinus* spp.). The larvae are gregarious and feed primarily on older needles in the summer (June-July in Fennoscandia). After finishing feeding, the larvae drop to the ground and spin a cocoon in the upper humus layer, often within the crown projection of the tree (Kolomiets et al. 1979). The larvae pupate within the cocoon and adults emerge after about 2 months (Wallace and Sullivan 1963). Sawfly cocoons for the experiment were collected from outbreak populations in southern Sweden by caging larvae in their later instars and allowing them to pupate and spin cocoons within the cage. Cocoons used in the temperature (laboratory) and microhabitat (field enclosure) experiments were collected in summer 2011 from one outbreak area (57°38′N, 15°93′E) while cocoons used in the alternative food (field-enclosure) experiment were collected in summer 2012 from another outbreak area (57°37′N, 16°14′E). In the experiments, the cocoons were mixed and randomly distributed between the experimental units. The cocoons were kept frozen to prevent the sawflies from hatching. A small study was conducted to investigate whether freezing affected the predation risk, and we found no difference in disappearance between previously frozen and non-frozen cocoons (χ^2^ = 0.78, *df* = 1, *p* = 0.38). In the method study, one frozen and one unfrozen cocoon were buried 1 cm in the soil at each side of a thin wooden stick at a distance of 1, 1.5, 2, 2.5 and 3 m from each of eight pine trees growing in a young stand (approximately 15 years) outside Uppsala, Sweden, i.e. 40 pairs of frozen and unfrozen cocoons. After 3 weeks the soil around the sticks was searched for cocoons and if not found they were considered predated.

The main predators in the pupal stage are small mammals, including voles and shrews, but birds and the larvae of click beetles (Elateridae) are also known predators. In our experiments we used bank voles (*Myodes glareolus* Schreber). The bank vole is the most common vole species in lowland forests in Fennoscandia. Their diet consists typically of vegetable matter but also includes animal material (Hansson 1985; Viro and Sulkava 1985). Like all endotherms, voles have a temperature optimum when the maintenance of body temperature requires least energy. The thermoneutral zone for bank voles is between 25 and 30 °C (Aalto et al. 1993). The experimental voles were born in the laboratory of the Suonenjoki research station of the Finnish Forest Research Institute (FFRI), and housed for 3–8 months in groups of two to four in maintenance cages (60 × 38 × 20 cm; Tecniplast, Italy) prior to the experiments. During the maintenance period, voles were provided with rodent laboratory pellets (Altromin 1314F; Altromin Spezialfutter, Germany) and water ad libitum, and turnip and apple pieces intermittently. All voles selected for the experiments had a body mass in the range 20–30 g, and represented a roughly equal sex ratio. For each replicate in all three experiments a new vole was used, i.e. each vole individual was only used once.

### Temperature experiment

To evaluate the effect of temperature on bank vole predation rates of sawfly pupae, a laboratory experiment was set up in a climate room at the FFRI Suonenjoki field research station in Finland (62°38′N, 27°7′E). The temperature was kept constant at either 15 or 20 °C, with a light period of 16 h light, 8 h dark. The lower temperature treatment was chosen to reflect the daily mean air temperature for the period that sawflies are naturally pupae. The 20 °C temperature treatment was chosen to reflect exceptionally warm temperatures.

Experimental cages (same specifications as the maintenance cages) were fitted with a chipboard tray with 24 nails (7 cm) protruding upwards from the bottom, spaced evenly in a 4 × 6 configuration at 6-cm intervals. The tray was covered with a 2-cm layer of sand, onto which 24 cocoons were placed singly at the base of each nail to facilitate relocation. All cocoons were then covered with a 2-cm layer of damp peat. An additional two cocoons were placed centrally on top of the peat to condition the voles to prey on them. A water bottle was attached to the cage and cotton wool added to provide nesting material.

The voles were placed singly in smaller cages (43 × 26 × 15 cm; Tecniplast) in the climate room 5 days before the experiment took place in order to acclimatise them to their new environment. During the acclimatization period the voles had free access to food (pellets and turnip) and water but when the experiment started and the voles were transferred to the experimental cage, this food was taken away. The voles were left in the experimental cages for 22 h (i.e. one feeding trial) and were then removed and transferred back into their maintenance cages. Cocoons were thereafter categorised as intact or empty. The experiment was repeated over four feeding trials for each temperature with six to 12 cages at a time; in total there were 34 replicates at 15 °C and 42 replicates at 20 °C.

### Microhabitat experiment

**Fig. 1:**
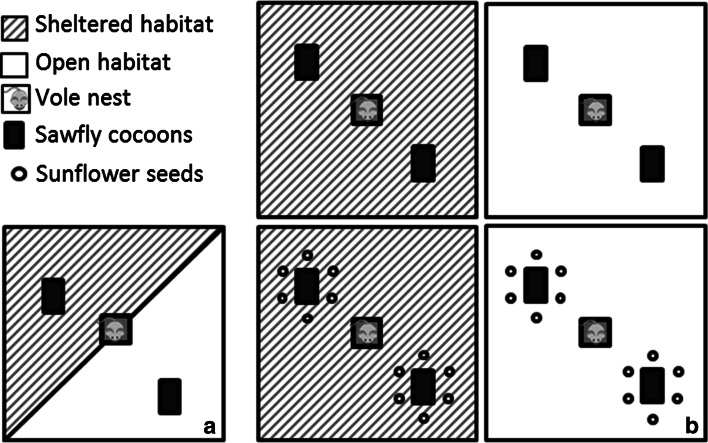
Schematic view of the study design for estimating pupal predation of *Neodiprion sertifer* by bank voles. In the microhabitat experiment the bank voles had access to both a sheltered and an open microhabitat (**a**). In the alternative food experiment the voles were assigned to one of the microhabitats, with or without access to sunflower seeds (**b**)

The effect of microhabitat on the pupal predation by voles was investigated in 3 × 3-m outdoor enclosures at the FFRI Suonenjoki field research station in Finland (62°38′N, 27°7′E). The enclosures were constructed of sheet metal extending 60 cm both below and above ground and protected with a 10-cm-mesh plastic net to prevent bird predation. A sheet metal shelter (40 × 40 × 40 cm) was located in the middle of each enclosure, and voles had access to this at all time. Half the area of each enclosure was prepared so that it was open and hostile; this was achieved by spreading a layer of soil over the short cut grass (to ca. 5-cm height) that covered the ground within the enclosure. In the other half of the enclosure branches and hay were spread out (over approximately two-thirds of the enclosure floor) to represent a rich habitat with a lot of structures providing shelter. One bank vole of random sex was placed into each enclosure 2 days before the experiment started, together with 0.5 dl of laboratory pellets. The experiment began by first removing all remaining pellets and thereafter placing two trays with 24 nails (like the ones in the temperature experiment) prepared with the same number of cocoons, one in each of the artificial microhabitats (Fig. 1a). The tray in the sheltered area was covered by a 2-mm metal wire mesh resting on the nails to enable branches to be placed over the tray without disturbing the tray surface. The voles were allowed to feed for 22 h. After the experimental period, the trays were collected and cocoons were categorised as intact or empty, as in the temperature experiment. We also added a third category: lost cocoons, i.e. ones that we could not find. In the field small mammals commonly move the cocoons from where found to store them elsewhere for later consumption (Buckner 1955). Lost cocoons together with the cocoons categorised as empty, were considered to be predated. The experiment was repeated in two feeding trials, on 16 and 21 September 2011. In the first trial, ten enclosures were used and in the second, 12.

### Alternative food experiment

The effect of alternative food was investigated in the same outdoor enclosures as in the microhabitat experiment. The difference was that for this experiment an enclosure was either completely open or completely sheltered, and either with or without an alternative food source (i.e. there were four treatments; Fig. 1b). Cocoons were presented in two trays per enclosure as in the microhabitat experiment. The alternative food, sunflower seeds, was presented in six Petri dishes (diameter 90 mm) spread evenly around the enclosure. Each dish contained four sunflower seeds (a total of 24 seeds per enclosure) laid on a layer of sand and lightly covered with a thin layer of peat. The voles were acclimatised in the enclosures for 2 days before the experiment was started, as in the microhabitat experiment. The voles were allowed to feed on the pupae for 19 h, after which intact, empty and lost cocoons were recorded, as were remaining seeds. The experiment was repeated in three feeding trials, beginning on 2, 6 and 15 October 2012. In the first trial 18 enclosures were used with four to five replicates of each treatment, in the second trial 15 enclosures were used with two to five replicates per treatment and in the third trial 14 enclosures were used with three to four replicates per treatment. The reason for the uneven replicate sizes was that at some occasions the vole had died and those replicates were therefore not included.

### Data analysis

To evaluate the effect of temperature on predation rate, the probability of the pupae being eaten was analysed. To quantify the effect of microhabitat and alternative food on predation rate, the probabilities of the pupae being eaten or lost were first analysed separately and then added together for analysis, since both empty and lost cocoons were considered to be predated. In each analysis, numbers of empty, lost or predated cocoons were used as binary variables (e.g. empty cocoons, not empty cocoons) and analysed with logistic regression models with logit link in the R software [the generalised linear model (glm) function, version 2.15.1; R Development Core Team 2012]. Due to overdispersion in the data (residual deviance larger than residual *df*) a quasi-binomial distribution was used. In the temperature experiment, temperature and vole sex were used as the explanatory variables in the full model. In the microhabitat experiment, habitat (sheltered/open area), vole sex and trial were used as explanatory variables in the full model. In the alternative food experiment, access to alternative food (yes/no), habitat (sheltered/open), vole sex and trial were used as explanatory variables in the full model. Trial was treated as a fixed factor since the number of trials (two and three, respectively) is too low to be meaningful as a random effect. Explanatory terms in the models were reduced by backward selection starting from the full model with interactions, dropping non-significant terms (at *α* = 0.05) one by one until the model only contained significant effects.

## Results

### Temperature experiment

**Fig. 2:**
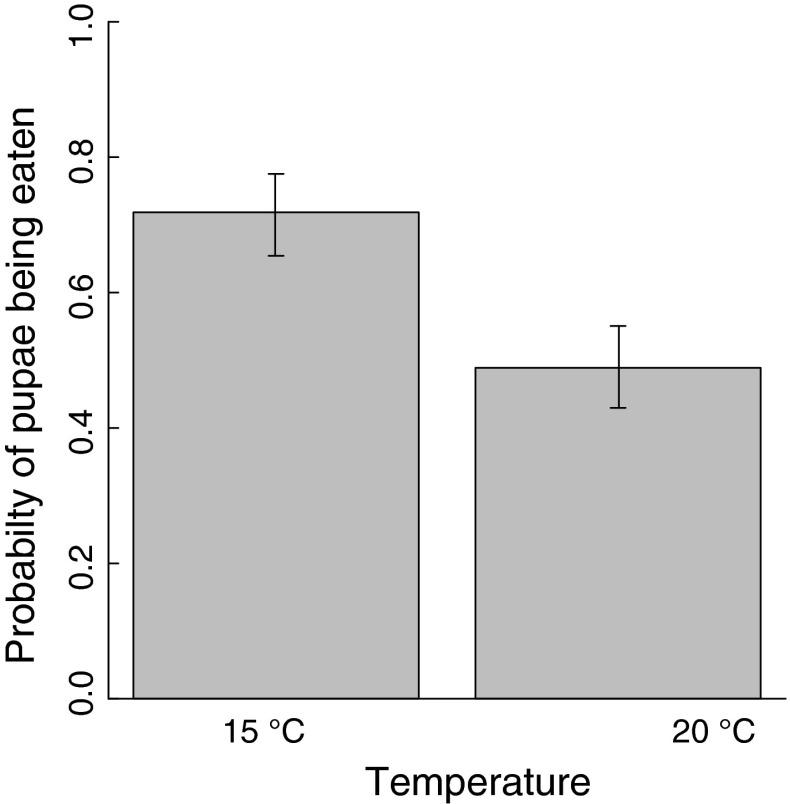
The probability (±SE) of sawfly pupae (*Neodiprion sertifer*) being eaten by bank voles at 15 and 20 °C

More pupae were eaten at 15 °C than at 20 °C (Fig. 2). The probability (±SE) of a pupa being eaten by a bank vole was 0.72 (0.65–0.78) at 15 °C compared to 0.49 (0.43–0.55) at 20 °C (likelihood-ratio χ^2^ = 6.7, *n* = 76, *p* = 0.01).

### Microhabitat experiment

**Fig. 3:**
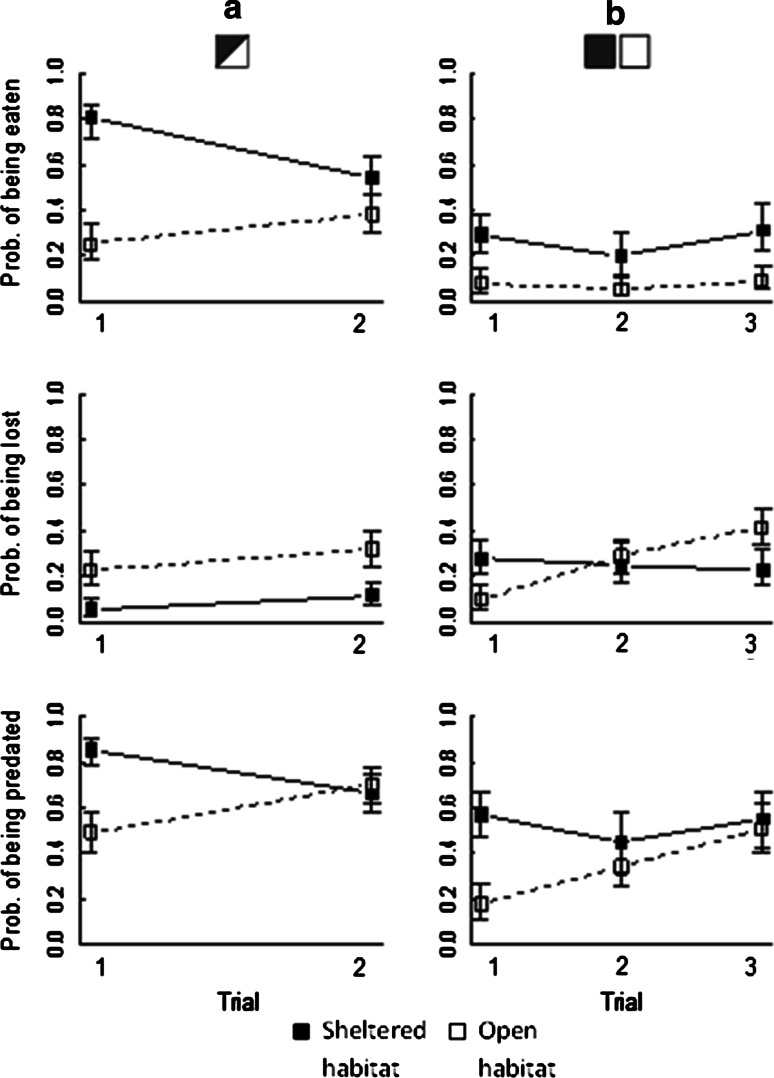
The probability (±SE) of sawfly pupae (*Neodiprion sertifer*) being eaten, lost or predated by bank voles in different microhabitats (covered or open area) and feeding trials (*1*, *2*,* 3*). Predated pupae were either eaten in situ or lost.* Left-hand figure parts* (**a**) describe feeding behaviour when the voles could choose between the covered and open areas; *right-hand figure parts* (**b**) show data relating to a situation where the voles had no choice

In the sheltered area 75 ± 6 % (mean ± SE) of the cocoons were either empty or lost as compared to 60 ± 6 % in the open area. However, the outcome was influenced by trial (Table 1; Fig. 3a). In the first trial the probability of being predated was higher in the sheltered area (separate GLM for trial 1; *t* = −3.6, *df* = 19, *p* = 0.002) whereas in the second trial there was no difference (separate GLM for trial 2; *t* = 0.3, *df* = 23, *p* = 0.76). The lowest probability of being predated (0.49) was found in the open area during the first trial. In the sheltered area most of the pupae (82–93 %) were eaten by the voles in situ, while in the open area half of the pupae (46–48 %) were removed from the unsheltered area to be consumed elsewhere (Table 1; Fig. 3a).

**Table 1:** Analysis of deviance table for generalised linear models of the probability of *Neodiprion sertifer* pupae being eaten, lost or predated (i.e. eaten + lost) by bank voles from a microhabitat experiment

	*χ* ^2^	*df*	*P*-value
Eaten			
Habitat	15.48	1	<0.001
Trial	0.57	1	0.45
Habitat × trial	5.5	1	0.02
Lost			
Habitat	9.88	1	<0.01
Predated			
Habitat	3.30	1	0.07
Trial	0.02	1	0.88
Habitat × trial	6.59	1	0.01

Explanatory variables in the full models were microhabitat (open/sheltered), feeding trial and vole sex. Results presented are from reduced models, i.e. if the interaction term was significant values are presented for all variables, if not values are only presented for significant main factors

The weather conditions differed between the trials. The daily mean temperature was 11.2 °C and there was a total rainfall of 0.4 mm during the first trial; during the second trial the mean temperature was 10.2 °C but the total rainfall amounted to 13.2 mm.

### Alternative food experiment

In 19 out of 24 enclosures in which the sunflower seeds had been added, all the seeds were eaten from the petri dishes. However, access to alternative food did not affect predation on pupae (χ^2^ = 0.002, *df* = 1, *p* = 0.96). In the sheltered habitat 53 ± 7 % (mean ± SE) of the pupae (i.e. eaten and lost cocoons combined) were predated compared to only 34 ± 6 % in the open habitat (Table 2; Fig. 3b). Of the predated pupae, 51 % were eaten in situ in the sheltered microhabitat compared to 21 % in the open area. There was no general effect of habitat type on the number of lost cocoons. However, the number of cocoons that were removed in the open habitat but not in the covered differed between trials (Table 2; Fig. 3b).

**Table 2:** Analysis of deviance table for generalised linear models of the probability of *Neodiprion sertifer* pupae being eaten, lost or predated (i.e. eaten + lost) by bank voles from an alternative food experiment

	*χ* ^2^	*df*	*P* value
Eaten			
Habitat	12.7	1	<0.001
Lost			
Habitat	0.01	1	0.93
Trial	3.95	2	0.14
Habitat × trial	6.82	2	0.03
Predated			
Habitat	4.38	1	0.04

Explanatory variables in the full models were microhabitat (open/sheltered), feeding trial, access to alternative food (yes/no) and vole sex. Results presented are from reduced models, i.e. if the interaction term was significant values are presented for all variables, if not values are only presented for significant main factors

During the first trial in the alternative food experiment, the daily mean temperature was 10.8 °C and the sky mostly clear (1.8-mm rainfall); during the second trial, the temperature was 6.7 °C and there was 6.9 mm of rain; during the third trial the temperature was 4.4 °C and there was 13.4 mm of rain.

## Discussion

The results suggest that predation of pine sawfly pupae by voles is lower at higher temperatures and in barren forests due to physiological and behavioural causes affecting the functional response, and not necessarily solely through effects on predator densities as previously proposed (Hanski and Parviainen 1985; Hanski 1987). More specifically, the temperature experiment in this study clearly showed that voles preyed less on *N. sertifer* pupae when exposed to the warmer temperature treatment (20 °C). This is expected, because small mammals respond to an increase in temperature by lowering their metabolism which reduces their demand for food (Sibly 1981). Small mammals have been shown to have lower reproductive success and, as a consequence, lower population densities during warm and dry summers (Pankakoski 1985; Lewellen and Vessey 1998). The occurrence of pine sawfly outbreaks has been explained by such decreases in small mammal populations, which leads to weaker top-down control (Hanski and Parviainen 1985). Our results indicate that also reduced predation pressure on pupae in warmer temperatures, regardless of changes in small mammal density, could add to a higher survival of pine sawflies, contributing to the initiation of an outbreak.

The predation risk-allocation hypothesis (Lima and Bednekoff 1999), supported by most studies on the topic (Verdolin 2006), states that animals should forage more in low-risk situations than in high-risk situations. Animals should stop foraging in a patch when the harvesting benefits fall below the energetic costs and the risk of being killed (Charnov 1976; Brown 1988). Only when the urge for food becomes great enough would animals be forced to forage in high-risk situations or locations. In our outdoor experiments in which we had simulated low- and high-risk situations, we found support for this hypothesis, although the differences in predation rates between the covered and open areas were smaller than expected. When given a choice, as in the microhabitat experiment, one would expect voles to deplete the resources in the sheltered habitat before venturing out into the risky open area (e.g. Kotler et al. 1991; Jacob and Brown 2000). It is plausible that the relative proximity of cocoons in the open area to the covered area led voles not to experience the open area, a high-risk habitat. However, the voles behaved differently in the different microhabitats. In the covered area more pupae were eaten in situ whereas in the open area more cocoons were removed. In a situation when the voles did not have a choice, as in the alternative food experiment, there was no general difference in the number of cocoons removed between habitats.

Although the outdoor experiment was not designed to study the effects of climatic factors on pupae predation rates, our results do identify weather as a potentially important factor. Interestingly, and for reasons that we can only speculate about, trial was a significant factor in both outdoor experiments. It is likely that weather during experimental trials influence the behaviour of voles (Doucet and Bider 1974; Vickery and Rivest 1992), and that rain has a greater effect than temperature. During trials with clear sky, pupal predation was higher in the covered area compared to the open area, whereas during trials with rain there was no difference. Our interpretation of these results is that a sheltered habitat is always a relatively good habitat for voles to feed in but that weather (i.e. precipitation) influences the propensity of voles to enter open habitats for foraging. Voles may feel more secure during rainfall because their natural enemies (e.g. birds of prey and weasels) are less active and/or efficient when it rains or when the illumination decreases due to cloud cover (Kotler et al. 1991). If so, weather in combination with habitat, may have a strong impact on pupal predation and deserve further studies.

According to optimal foraging theory, the best strategy for a generalist predator would be to specialise on, and whenever necessary switch to, the most profitable food source (Stephens and Krebs 1986). The role of alternative food as an influential component of population dynamics in predator–prey systems has received much attention (Murdoch 1969; Angelstam et al. 1984). In general, pupal predation per individual may decrease when small mammals are offered an alternative food source of higher quality (Murdoch 1969; Elkinton et al. 2004). We did not find support for the hypothesis that, in the open habitat, pupal predation would decrease more than in the sheltered habitat when alternative food in the form of seeds were also available. In fact, there was no difference in the number of predated pupae between enclosures with or without seeds. Although the relative preference of voles for seeds vs. pupae is not established, in most of the cases all the seeds had been eaten, suggesting vole preference for the seeds. Probably, the voles concentrated on these before searching for the pupae. In the ‘rich’ habitat however, voles ate more food in total, since they consumed both pupae and seeds. A high diversity and abundance of food within a vole’s home range may enhance their feeding activity.

The cause of outbreaks in forest insect pests might be explained by weakened top-down predatory control due to decreases in small mammal population densities caused by weather, microhabitat and/or food supply (Hanski and Parviainen 1985). In this study we demonstrate that each of these factors also can explain variation in predator behaviour and predation rates without there being changes in predator densities. In combination with temperature-triggered physiological responses, such as altered metabolism, changes in behaviour related to microhabitat could be a potential contributor to the onset of outbreaks of defoliators which pupate in the ground. We acknowledge, however, that other factors interacting with forest insects may be affected by weather and microhabitat and thereby play a role for outbreak dynamics, for example host plant quality (White 1974; Larsson and Tenow 1984; Mattson and Haack 1987). In general, our ability to predict future events such as insect outbreaks largely depends on how well we understand mechanistic interactions between different trophic levels and the dynamics of populations and communities. The results presented here highlight that in order to relate observed patterns in prey population dynamics in nature to putative mechanisms, involving natural enemies, one needs to evaluate not only changes in densities but also behavioural responses of the enemies to the biotic and abiotic environment.
